# Hemolysis as a rare but potentially life-threatening complication of hemodialysis: a case report

**DOI:** 10.1186/1756-0500-7-475

**Published:** 2014-07-28

**Authors:** Jenni Yoon, Sritika Thapa, Robert Dobbin Chow, Bernard G Jaar

**Affiliations:** 1 Department of Medicine, MedStar Good Samaritan Hospital, 5601 Loch Raven Boulevard, Suite 3 North, Baltimore, MD, USA; 2 Department of Medicine, Johns Hopkins School of Medicine, Baltimore, MD, USA; 3 Department of Epidemiology, Johns Hopkins Bloomberg School of Public Health, Baltimore, MD, USA; 4 Welch Center for Prevention, Epidemiology and Clinical Research, Johns Hopkins Medical Institutions, Baltimore, MD, USA; 5 Nephrology Center of Maryland, Baltimore, MD, USA

**Keywords:** Hemolysis, Hemodialysis, Anemia, Complications, Hyperkalemia, Myocardial infarction

## Abstract

**Background:**

The burden of end-stage renal disease (ESRD) in the United States has increased dramatically over the past 30 years with almost 613,000 patients receiving renal replacement therapy in 2011. That same year, more than 112,000 new patients initiated dialysis with 92% of them receiving hemodialysis (HD). These patients experience significant morbidity and mortality with very frequent emergency room visits. Acute hemolysis associated with HD is a rare complication; however, if it’s not recognized early and managed adequately, it can be associated with life-threatening complications such as hyperkalemia and even myocardial infarction.

**Case presentation:**

66-year-old African-American female with a history of ESRD secondary to hypertension developed a blood infiltration on the arterial side of her arteriovenous fistula followed by sudden onset of diffuse abdominal pain with nausea and vomiting during her regular HD treatment. She was referred to the emergency department where she was found to have shortness of breath with improved gastrointestinal symptoms. Her initial work-up revealed a severe anemia with a hematocrit of 10%. Further work-up revealed massive hemolysis, likely mechanical in nature and believed to be induced by malpositioning of her HD needle in the fistula. Her hospital course was complicated by rhabdomyolysis and acute myocardial infarction thought to be secondary to supply–demand ischemia in the setting of her profound anemia. Within a week, she eventually had a full recovery.

**Conclusion:**

It is extremely important for physicians and particularly emergency department physicians to be aware of this potentially life-threatening complication of HD and have a high index of suspicion in the setting of acute anemia with hemolysis in this population.

## Background

The burden of ESRD in the United States has increased dramatically over the past 30 years with almost 613,000 patients receiving renal replacement therapy in 2011. That same year, more than 112,000 new patients initiated dialysis with 92% of them receiving HD [1]. These patients experience significant morbidity and mortality with very frequent emergency room visits. Acute hemolysis associated with HD is a rare complication; however, if it’s not recognized early and managed adequately, it can be associated with life-threatening complications such as hyperkalemia and even myocardial infarction [2].

## Case presentation

A 66-year-old African-American female with a two-year history of ESRD presumed secondary to longstanding hypertension was in her usual state of health until her HD treatment on the day of admission. She is known to have well-controlled anemia of chronic kidney disease treated with darbepoetin alfa without iron deficiency. Her high blood pressure has been stable on metoprolol tartrate and nifedipine. She had one prior history of congestive heart failure in the setting of fluid overload but no documented history of atherosclerotic cardiovascular disease. She took sevelamer carbonate regularly with her meals to control her hyperphosphatemia. No new medications were added to her medical regimen over the past several weeks prior to her hospitalization. On the day of admission, she had a routine HD treatment start; however within two hours into her regular HD session, she developed sudden onset of diffuse abdominal pain, associated with nausea, and vomiting. Although reportedly the patient remained hemodynamically stable, the dialysis session was immediately interrupted and she was found to have blood infiltration around the arterial needle insertion site of her left upper arm arteriovenous fistula. Her symptoms resolved spontaneously and she went home with her daughter. Within the hour and while at home, the patient started feeling very weak, nauseated and had new onset of red colored urine. Subsequently, she became cyanotic with shortness of breath. Because of these symptoms, emergency medical services were called and she was brought to our emergency department.

During her initial evaluation, she was afebrile and her vital signs were significant for a blood pressure of 154/86 mmHg, a heart rate of 96 beats per minute, respiratory rate of 20 breaths per minute, and oxygen saturation of 97% on 3 liters of oxygen via nasal cannula. Her physical examination was otherwise unremarkable except for bruising and hematoma 2 cm × 2.5 cm over her HD vascular access. Many of the laboratory values obtained in the emergency room were initially limited due to interference (Table 1). However, her hematocrit was 10%, with a platelet count of 487,000 per microliter. During her follow-up work-up, the hematocrit level was confirmed to be 10% but the platelet count dropped significantly to 79,000 per microliter and her peripheral smear showed many schistocytes. The patient hemolytic panel was consistent with hemolysis; haptoglobin was less than 30 mg/dL; fibrin split products greater than 20 g/mL and a lactate dehydrogenase of 1,722 IU/L. Prothrombin time and partial thromboplastin time were normal. Her serum potassium was 4 mmol/L. Her fecal occult blood test was negative. Thrombotic thrombocytopenic purpura was considered in the differential diagnosis but ruled out due to the patient’s appropriate response to blood transfusion therapy and lack of neurologic symptoms. Given the patient’s elevated myoglobin, which peaked at 3,101 ng/ml, a rheumatologic workup was ordered and found to be normal. A muscle biopsy showed scattered round, polygonal and angular atrophic myofibers consistent with nonspecific myofiber atrophy. Urine porphobilinogen, serum copper and aluminum levels were all within normal limits. Blood and urine cultures were negative. The patient received packed red blood cells and her hemoglobin and hematocrit rose appropriately. Her hospital course was complicated by an acute myocardial infarction thought to be secondary to supply–demand ischemia in the setting of her profound anemia. She eventually had a full recovery and was discharged from the hospital within one week. Subsequent dialysis sessions were managed with extra care and were uneventful.

**Table 1:** Timeline of pertinent laboratory data

	**0 hour**	**5 hours**	**48 hours**	**Day 5**	**Day 10**
Hemoglobin (gm/dL)	*	*	12.1	11.1	10.4
Hematocrit (%)	10	10	33.3	33.8	30.6
Platelet (microliter)	487,000	79,000	clumping	80,000	196,000
Lactate dehydrogenase (IU/L)	N/A	*	1722	664	N/A
Haptoglobin (mg/dL)	N/A	*	<31	120	N/A

*Interference of laboratory values was a result of free hemoglobin producing a red discoloration of the serum even after centrifugation.

N/A: not available. Test was not ordered at that time.

Our patient experienced a severe case of hemolysis associated with HD. This is an uncommon complication of HD but it can be associated with significant morbidity and even death if not recognized early. Hemolysis in this context may be a consequence of a number of biochemical, toxic and mechanical complications during the HD procedure itself. Four major mechanisms capable of inducing hemolysis during HD have been recognized: overheating, hypotonicity of the dialysate, contamination of the dialysate with toxins (e.g., formaldehyde, bleach, chloramine, nitrates) from the water supply, with copper from copper piping and improperly functioning clamps or tubing, such as kinked arterial lines [3-6]. In cases where there is a contamination of the water supply, most of the patients in the concurrent dialysis session can experience similar signs and symptoms associated with hemolysis [4]. Toxins can also include contamination of the dialysate with bacteria, endotoxins, and disinfectants although hemolysis due to these toxins is very rare. In one simulated HD study where post HD blood samples were run through faulty tubing, gross hemolysis occurred within thirty minutes [7].

If faulty tubing was the culprit, one would expect to find sporadic events linked to the manufacturer of these supplies. Further, if biochemical or toxic causes were associated with this event, one would expect to see other patients with similar symptoms and hemolysis. Kinked HD blood lines have been associated with sporadic episodes of hemolysis during HD. However, we believe that this unfortunate event in our patient was induced by malpositioning of the needle on the arteriovenous fistula, particularly given the abrupt onset of symptoms within hours after starting HD, absence of kinked blood lines at the time of the event, presence of blood infiltration with hematoma at the needle insertion site and that this was again a completely isolated event since no other patients suffered this complication. This hemolytic phenomenon is sometimes called “red cell fragmentation syndrome” because the hemolysis is in large part due to intravascular mechanical injury with destruction of the red blood cells.

Our patient presented with a critical hematocrit level; this seems unique among most HD induced hemolysis cases reported. Other case reports have usually described only minor decrease in hematocrit. This major hemolytic event with hypoxemia may explain the cause of her elevated myoglobin and acute coronary syndrome, which to our knowledge has not been described in other HD-induced hemolysis cases reported. The interference of laboratory values was a result of free hemoglobin that produced a red discoloration of the serum (known as port wine) even after centrifugation. Our patient serum potassium was not elevated, at least in part because the dialysis staff did not reinfuse the patient’s blood back after stopping the HD treatment.

## Conclusions

The diagnosis of HD-induced hemolysis requires a high level of suspicion. If hemolysis is suspected during HD, the patient’s blood in the extracorporeal dialysis circuit should not be reinfused and should be discarded. This is an important step to lower the risk of hemolysis related complications such as hyperkalemia and related arrhythmias. In all cases, these patients should be referred to the emergency department immediately after stopping HD for further management including close monitoring of hemoglobin and serum potassium since most HD patients in the United States are treated in free standing dialysis units where there are no clinicians (physicians and physician extenders) on site except during their dialysis rounds and no medical laboratory on site. Similarly in other countries where HD sessions are sometimes performed in the absence of a nephrologist, HD emergencies may be handled by any other available physicians [8].

Given the serious nature, potentially life-threatening, of HD-induced hemolysis, it is imperative for all physicians and particularly emergency department physicians to keep this possible diagnosis high in their differential diagnosis, especially in the presence of acutely worsening anemia. The most common presenting symptoms are non-specific and may include nausea, vomiting, and abdominal pain. Other symptoms may be shortness of breath, chest pain, back pain and headaches. On physical examination, some patients have been found to have worsening hypertension [6]. Differential diagnoses for HD-induced hemolysis include hemorrhage, thrombotic thrombocytopenic purpura, infection, autoimmune process, and drug reactions.

In summary, HD-induced hemolysis requires immediate recognition and management (Table 2); thus the importance of dialysis staff but also emergency department physicians awareness. Initial work-up in the emergency department should rule out hemolysis, assess need for blood transfusion, monitoring and treatment of hyperkalemia as clinically indicated [2,9]. These steps are vital, as the severity of this complication can be catastrophic and rapidly life threatening.

**Table 2:** Clinical pearls of hemodialysis-induced hemolysis for the emergency medicine physician

1. Presentation:	● Hemodialysis–induced hemolysis can be very subtle
	● If a dialysis patient presents acutely during hemodialysis treatment with nausea, vomiting, and abdominal pain, be sure to have a high suspicion for this condition
2. Recognition:	● Emergency department physicians need to be aware of this hemodialysis complication in order to begin timely management
3. Differentials:	● Mechanical vs. Chemical
	● Think of the 3 C’s: Chlorine, Copper, and Chloramine
4. Intervention:	In the hemodialysis unit:
	● tubing must be clamped immediately and not returned to the patient given the risk of hyperkalemia
	In the emergency department:
	● Confirm hemolysis
	● Prepare for possible blood transfusion
	● Check serum potassium
	Prepare for hyperkalemia treatment, including hemodialysis

## Consent

Written informed consent was obtained from the patient for publication of this Case Report and any accompanying images. A copy of the written consent is available for review by the Editor-in-Chief of this journal.

## Abbreviations

ESRD: End-stage renal disease; HD: Hemodialysis.

## Competing interests

The authors declare that they have no competing interests.

## Authors’ contributions

JY: has made substantial contributions to conception and design and acquisition of data, involved with drafting the manuscript, has given final approval of the version to be published. ST: has made substantial contributions to acquisition of data, involved in revising the manuscript critically for important intellectual content and has given final approval of the version to be published. RDC: has made substantial contributions to acquisition of data, involved in revising the manuscript critically for important intellectual content and has given final approval of the version to be published. BGJ: has made substantial contributions to conception and design and acquisition of data, involved with drafting the manuscript, revising the manuscript critically for important intellectual content and has given final approval of the version to be published. All authors read and approved the final manuscript.
